# Carbohydrate antigen 125 for mortality risk prediction following acute myocardial infarction

**DOI:** 10.1038/s41598-020-67548-8

**Published:** 2020-07-03

**Authors:** Felipe Falcão, Flávio Oliveira, Fabiano Cantarelli, Rodigo Cantarelli, Paulo Brito Júnior, Hygor Lemos, Paloma Silva, Irla Camboim, Maria Cleide Freire, Osmário Carvalho, Dário Celestino Sobral Filho

**Affiliations:** 1 Departamento de Medicina Interna, Universidade de Pernambuco (UPE), Garanhuns, PE Brazil; 2 Unidade de Cardiologia Invasiva (UCI) – Hospital Memorial São José, Rede d’Or São Luiz, Recife, PE Brazil; 3 Pronto Socorro Cardiológico de Pernambuco (PROCAPE-UPE), Recife, PE Brazil

**Keywords:** Biomarkers, Cardiology, Interventional cardiology

## Abstract

Carbohydrate antigen 125 (CA125) is a congestion and inflammation biomarker and has been proved to be related to a worse prognosis in heart diseases. However, the precise relationship between elevated CA125 in patients with ST-segment elevation myocardial infarction (STEMI) has not yet been sufficiently studied. We set out to determine the association of CA125 with all-cause mortality at 6 months in STEMI. CA125, N-terminal pro brain natriuretic peptide (NTproBNP) and high sensitive C-reactive protein (hs-CRP) were measured in 245 patients admitted consecutively with STEMI undergoing coronary angioplasty. The mean age in our sample was 63.7 years, 64.9% were males, 28.3% had diabetes and 17.7% presented with acute heart failure (Killip ≥ 2). The median serum level of CA125 was 8.1 U/ml. At 6 months, the rate of all-cause mortality was 18% (44 patients). Receiver operating characteristic curve analysis demonstrated that CA125 presented similar performance to predict mortality as NTproBNP and hs-CRP. Patients with CA125 ≥ 11.48 had a higher rate of mortality (Hazard Ratio = 2.07, 95% confidence interval = 1.13–3.77, *p* = 0.017) than patients with CA125 < 11.48. This study suggests that elevated CA125 levels might be used to identify patients with STEMI with a higher risk of death at 6 months. CA125 seems to be a similar predictor of mortality compared to NTproBNP and hs-CRP.

## Introduction

Myocardial infarction with ST-segment elevation (STEMI) is a life-threatening disorder with high morbidity and mortality despite advances in treatment. Patients presenting with STEMI tend to be heterogeneous and immediate risk stratification at the time of presentation is essential for optimal management^1–4^.

Markers of congestion and inflammation, such as natriuretic peptides (NP) and high sensitive C-reactive protein (hs-CRP), have been shown to be prognostic markers. However, the biomarkers currently available are not perfect, and their correct interpretation requires careful consideration of the specific clinical scenario^5^.

Carbohydrate antigen 125 (CA125) is a congestion and inflammation biomarker. It has been studied in patients with heart diseases, especially heart failure^6^. However, the precise relationship between elevated CA125 in patients with STEMI and cardiovascular events has not yet been sufficiently studied. This study set out to evaluate the relationship between CA125 and mortality in STEMI patients in comparison with N-Terminal pro brain natriuretic peptide (NTproBNP) and hs-CRP.

## Methods

### Patients and study design

This was a prospective cohort at a single center. Patients consecutively admitted with STEMI undergoing primary angioplasty were included. The diagnosis of STEMI was made based on the third universal definition^7^. Two hundred and seventy one patients were considered to be included. Patients were excluded if they had chronic heart failure (n = 3), previous coronary revascularization (n = 11), kidney failure (n = 8), absence of severe coronary disease (n = 4), end-stage liver disease, ongoing infection or malignancy.

One experienced interventional cardiologist, who was blinded to the patient's clinical data, analyzed all quantitative coronary angiography data. Thrombolysis in myocardial infarction (TIMI) flow was assessed before and after the angioplasty^8^. Coronary artery disease was defined as a single vessel or multivessel disease, according to the number of epicardial arteries with at least one lesion measuring > 50% diameter stenosis. Procedural success was defined as achieving a minimum stenosis diameter reduction to less than 10% in the infarct-related artery, along with TIMI flow ≥ 2 with no angiographic complications^9^.

Coronary anatomy complexity was evaluated by the residual Syntax Score (rSS), which involves calculation of the Syntax Score after angioplasty^10^. This score evaluates stenosis that has caused a reduction of more than 50% in luminal diameter, in vessels measuring 1.5 mm or more in diameter. The number and extent of lesions, the tortuosity of the affected segments, the presence of thrombus or calcification, total occlusion, and involvement of bifurcation or trifurcation were evaluated. Each selected lesion was scored, according to its complexity. The SS is the sum of the individual scores for each lesion and was calculated using the SYNTAX Score Calculator software version 2.11 (SYNTAX Score Working Group, www.syntaxscore.com).

The choice of access route and type of stent was left entirely to the discretion of the operator.

The local ethics committee (*Instituto de Medicina Integral Prof. Fernando Figueira*) approved the study protocol, and all patients provided written informed consent to their participation. All the procedures were carried out in accordance with the relevant guidelines and regulations.

The outcome of the study was all-cause mortality at 6 months. Survival status was obtained by consulting the Mortality Information System of the Regional Department of Health.

### Laboratory measurements

Blood samples, taken preferentially in the hemodynamic ward from the moment that the access route was achieved for the angioplasty, were placed in vacutainer test tubes according to the manufacturer’s instructions. The blood samples were then centrifuged and stored at − 80 °C until the biomarker assays were performed. CA125, NTproBNP, and hs-CRP were determined using commercially available kits (Roche® Diagnostics). The manufacturer’s cutoff points for CA125, NT-proBNP and hs-CRP test result are 35 U/ml, 125 pg/ml and 0.5 mg/dl, respectively.

### Statistical analysis

Continuous variables were expressed as means and standard deviations or as the median and the 25th and 75th percentiles, according to the presence or absence of a normal distribution, as evaluated by the Kolmogorov–Smirnov test. The Mann–Whitney test and Student’s t-test were used for continuous variables according to their distribution. Categorical variables were expressed as absolute numbers and percentages. Fisher’s Exact test and Pearson’s chi-square test were used when appropriate. A “*p*” value of less than 0.05 was considered significant.

The receiver operating characteristic (ROC) curve was used to determine the greatest area under the curve (AUC) and the optimal cutoff value in predicting mortality of CA125, NTproBNP and hs-CRP. The AUC were compared with the DeLong test, using MedCalc Statistical Software version 18 (MedCalc Software bvba, Ostend, Belgium). All remaining statistical analyses were conducted using SPSS Statistics Software, version 21 (IBM, Armonk, NY, USA). The population was divided according to the optimal cutoff value for each marker and survival curves were generated by the Kaplan–Meier method. The log-rank test was used to evaluate the significance of differences between groups.

Univariate and multivariate regression analyses were used to identify potential independent predictors of mortality. Variables with “*p*” < 0.2 in bivariate analysis were included in the model. The variables included were age, gender, congestion, NTproBNP, CA125, hs-CRP, systolic and diastolic blood pressure, heart rate and rSS. The variables with “*p*” < 0.2 in the univariate regression were maintained in the model (age, congestion, NTproBNP, diastolic blood pressure and heart rate). The model was performed using backward stepwise selection and it was accepted (*p* < 0.001) and proved to be well adjusted according to the Lemeshow test (*p* = 0.824) as it correctly classified 85.6% of cases. Odds ratio (OR) and their respective confidence intervals (95% CI) were used to quantify the effects.

## Results

The mean age in our sample was 63.7 ± 13.1, 159 (64.9%) were males, 68 (28.3%) had diabetes and 41 (17.7%) presented with acute heart failure (Killip ≥ 2). Patients had a mean left ventricle ejection fraction (LVEF) of 54.2 ± 11.3%. The median serum level of CA125 was 8.1 (5.7–12.1) U/ml. The median values of NTproBNP and hs-CRP were 826 (202–2,374) pg/ml and 0.5 (0.25–1.76) md/dL, respectively (Table 1).

**Table 1:** Clinical characteristics of the entire population and according to all-cause mortality.

Variables	All patients N = 245	All-cause mortality		*p*
		No n = 201	Yes n = 44	
Age (years)	63.7 ± 13.1	62.5 ± 12.8	69.3 ± 13.4	0.002
Male	159 (64.9%)	134 (66.7%)	25 (56.8%)	0.215
Hypertension	161 (67.1%)	129 (65.5%)	32 (74.4%)	0.259
Diabetes	68 (28.3%)	53 (26.9%)	15 (34.9%)	0.293
Dyslipidemia	71 (29.6%)	57 (28.9%)	14 (32.6%)	0.637
Current smoker	85 (34.7%)	65 (32.3%)	20 (45.4%)	0.096
Family history of CAD	49 (20.4%)	42 (21.3%)	7 (16.3%)	0.458
Killip ≥ II	41 (17.7%)	18 (9.6%)	23 (53.5%)	< 0.001
GRACE score	114 (92–131)	110 (90–125)	141 (123.25–170.25)	< 0.001
Systolic blood pressure (mmHg)	133.3 ± 33.2	135.5 ± 31.4	124.5 ± 34.1	0.047
Diastolic blood pressure (mmHg)	82.7 ± 17.9	84.1 ± 17.6	77.3 ± 18.7	0.028
Heart rate	79.9 ± 19.8	78.0 ± 18.8	87.3 ± 21.9	0.007
LVEF	54.2 ± 11.3	55.2 ± 10.9	43.1 ± 10.1	0.001
Anterior STEMI	115 (47.1%)	91 (45.5%)	24 (54.5%)	0.276
Total ischemic time (min)	430 (290–625)	433 (291–629)	402 (263–512)	0.328
CA125 (IU/mL)	8.1 (5.7–12.1)	8.0 (5.5–11.2)	9.2 (6.4–14.5)	0.056
NTproBNP (pg/mL)	828.6 (202–2,374)	783.1 (184–2,117)	1,418.0 (302–7,714)	0.013
hs-CRP (mg/dL)	0.50 (0.25–1.76)	0.48 (0.22–1.45)	0.81 (0.36–5.57)	0.004

*CAD *coronary artery disease, *LVEF* left ventricle ejection fraction, *STEMI* ST-segment elevation myocardial infarction, *CA125* carbohydrate antigen 125, *NTproBNP* N-terminal pro brain natriuretic peptide, *hs-CRP* high sensitive C-reactive protein, *GRACE risk* global registry of acute coronary events.

After 6 months of follow-up, a total of 44 deaths had been identified: 19 during hospitalization and 25 at follow-up. Median age, Killip ≥ 2, arterial pressure (systolic and diastolic), heart rate and ejection fraction were the clinical variables that differed between the groups (Table 1). In addition, patients who died had a significantly lower incidence of angiographic success, a higher rSS and more multivessel disease (Table 2).

**Table 2:** Angiographic characteristics of the entire population and according to all-cause mortality.

Variables	All patients N = 245	All-cause mortality		*p*
		No n = 201	Yes n = 44	
**Initial TIMI flow**				
≤ 1	146 (75.6%)	111 (72.5%)	35 (87.5%)	0.050
≥ 2	47 (24.4%)	42 (27.5%)	5 (12.5%)	
Angiographic success	174 (90.2%)	144 (94.1%)	30 (75.0%)	< 0.001
Multivessel	119 (61.0%)	88 (57.1%)	31 (75.6%)	0.031
rSS	7.0 (0–14.3)	5.0 (0–12)	13.5 (5.2–18)	0.001

*rSS* residual Syntax Score.

CA125 when evaluated as a continuous variable showed a strong tendency towards statistical significance for mortality (9.2 [6.4–14.5] vs. 8.0 [5.5–11.2], *p* = 0.056). NTproBNP and hs-CRP levels were higher in patients who died.

**Figure 1:**
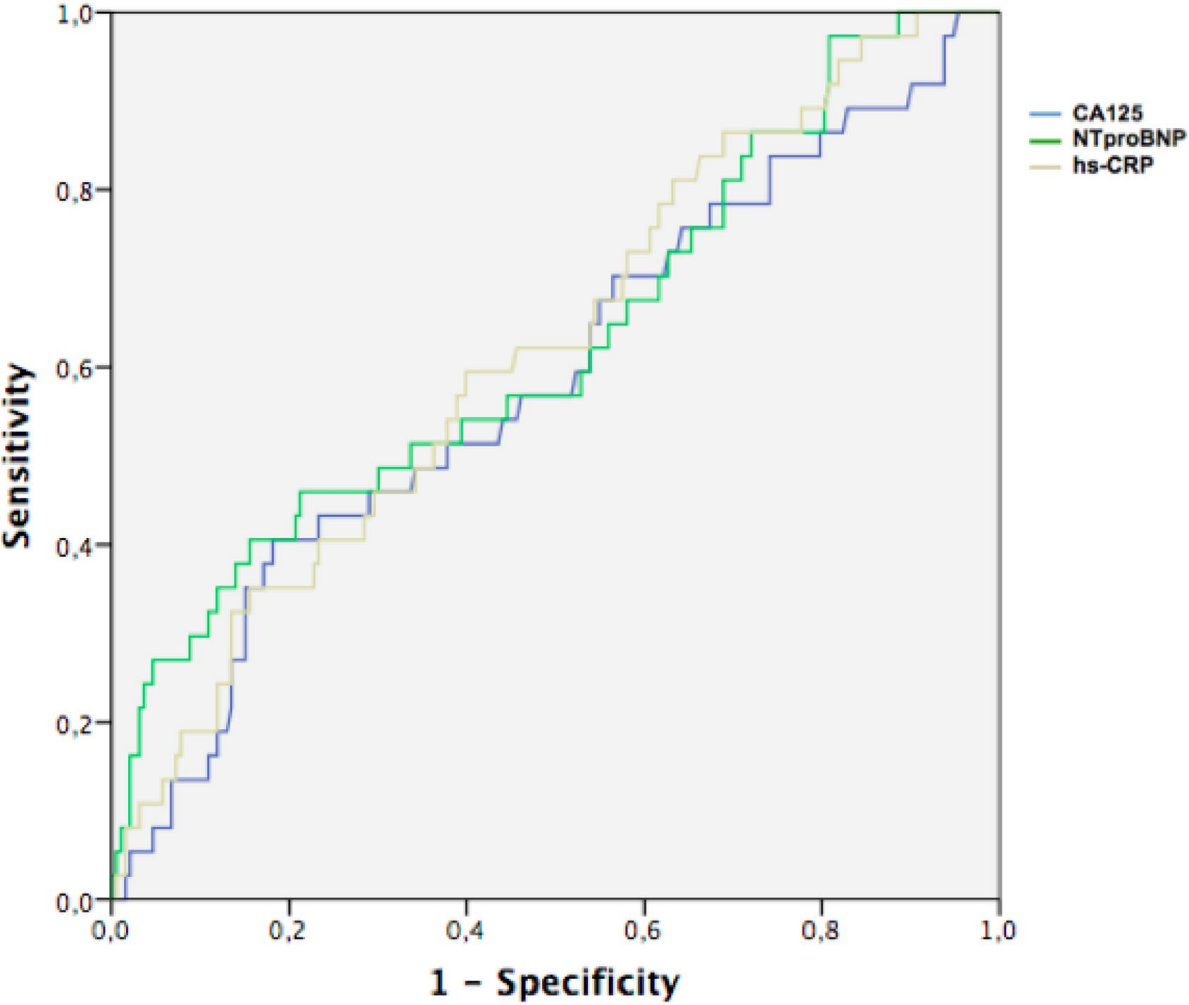
Receiver operating characteristic (ROC) curves comparing carbohydrate antigen 125 (CA125), N-terminal pro brain natriuretic peptide (NT-proBNP) and high sensitive C-reactive protein (hs-CRP) for prediction of 6 months all-cause mortality at 6 months.

Figure 1 demonstrated the ROC curves for each biomarker. The AUC for mortality were 0.589, 0.623 and 0.645 for CA125, NTproBNP and hs-CRP, respectively (Table 3). When AUC was compared two-by-two with the DeLong test, there was no statistically significant difference (Table 4).

**Table 3:** Area under the receiver operating characteristic (ROC) curves.

Biomarker	Area	CI 95%
CA125	0.589	0.516–0.659
NTproBNP	0.625	0.516–0.733
hs-CRP	0.631	0.533–0.729

*CI95%* confidence interval 95%, *CA125 *carbohydrate antigen 125, *NTproBNP* N-terminal pro brain natriuretic peptide, *hs-CRP* high sensitive C-reactive protein.

**Table 4:** Comparison of ROC curves based on the differences between the areas under de curves.

	hs-CRP	NTproBNP
CA125	0.0418 *p* = 0.569	0.0357 *p* = 0.619
NTproBNP	0.00611 *p* = 0.901	–

DeLong test. *CA125* carbohydrate antigen 125, *hs-CRP* high sensitive C-reactive protein, *NTproBNP* N-Terminal pro brain natriuretic peptide.

The optimal cutoff level of CA125, NTproBNP and hs-CRP were 11.48 IU/ml, 2348 pg/ml and 1.11 mg/dL, respectively. The performance in predicts mortality was similar between the three biomarkers (Table 5). Clinical and angiographic characteristics of the population according to CA125 cutoff are presented in Table 6. Patients with CA125 ≥ 11.48 had more acute heart failure and higher levels of GRACE score, hs-CRP, NTproBNP and mortality.

**Table 5:** Performances to predict mortality of CA125, NTproBNP and hs-CRP.

	Optimal cutoff	Sensitivity (%)	Specificity (%)	PPV (%)	NPV (%)
CA125	11.48	41	77	28	85
NTproBNP	2,348	44	80	30	87
hs-CRP	1.11	50	71	26	87

*CA125* carbohydrate antigen 125, *hs-CRP* high sensitive C-reactive protein, *NTproBNP* N-Terminal pro brain natriuretic peptide, *PPV* predictive positive value, *NPV* negative predictive value.

**Table 6:** Characteristics of the population according to CA125 levels.

Variables	CA125		*p*
	< 11.48 n = 181	≥ 11.48 n = 64	
Age (years)	62.77 ± 13.65	60.41 ± 12.29	0.202
Hypertension	147 (81.2%)	47 (73.4%)	0.189
Diabetes	51 (28.1%)	18 (28.1%)	0.985
Dyslipidemia	55 (30.3%)	17 (26.5%)	0.553
Current smoker	74 (40.8%)	21 (32.8%)	0.491
Killip ≥ II	23 (12.7%)	19 (29.6%)	0.004
GRACE score	143.5 ± 33.9	160.9 ± 43.8	0.003
Systolic blood pressure (mmHg)	134.9 ± 32.33	128.6 ± 31.6	0.235
Diastolic blood pressure (mmHg)	83.4 ± 18.1	80.73 ± 17.7	0.350
Heart rate	78.8 ± 17.8	83.2 ± 23.9	0.360
rSS	6 (0–14)	7 (1–15)	0.514
hs-CRP (mg/dL)	0.4 (0.2–1.3)	1.0 (0.4–3.2)	0.006
NTproBNP (pg/mL)	586.5 (170.3–1,812)	2089 (736.4–4,249)	< 0.001
Angiographic success	168 (92.8%)	53 (82.8%)	0.041
All-cause mortality	26 (14.3%)	18 (28.1%)	0.014

*CA125* carbohydrate antigen 125, *NTproBNP* N-terminal pro brain natriuretic peptide, *hs-CRP* high sensitive C-reactive protein, *GRACE risk* global registry of acute coronary events, *rSS* residual Syntax Score.

**Figure 2:**
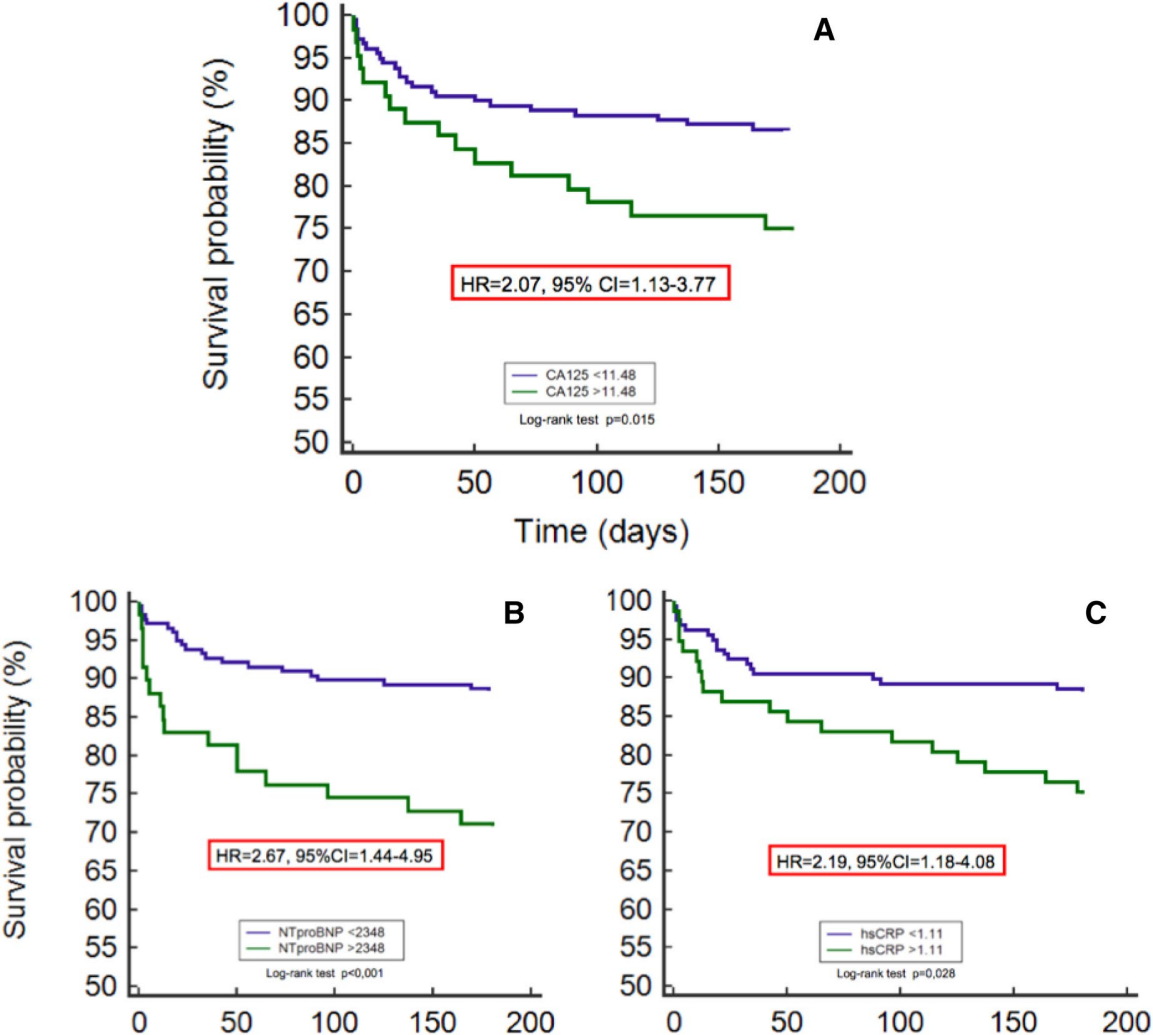
Survival probability according to elevated levels of CA125 (**A**), NTproBNP (**B**) and hs-CRP (**C**).

Patients with elevated values of CA125 (Hazard Ratio [HR] = 2.07, 95% confidence interval [CI] = 1.13–3.77, *p* = 0.017), NTproBNP (HR = 2.67, 95% CI = 1.44–4.95, *p* = 0.001) and hs-CRP (HR = 2.19, 95%CI = 1.18–4.08, *p* = 0.012) according to the optimal cutoff, had a worse prognosis. Kaplan–Meier cumulative survival curves are presented in Fig. 2.

Age, congestion, diastolic blood pressure and heart rate were independent predictors of mortality based on the multivariate regression analysis (Table 7).

**Table 7:** Independent predictors of mortality according to multivariate regression analysis.

Variable	Univariate		Multivariate	
	OR and 95% CI	*p*	OR and 95% CI	*p*
**Age**		0.002		0.001
< 70	1.00		1.00	
≥ 70	2.95 (1.48–5.87)		6.19 (2.05–18.60)	
**Congestion**		< 0.001		< 0.001
Yes	9.84 (4.55–21.25)		8.56 (2.86–25.56)	
No	1.00		1.00	
**NTproBNP**		0.002		0.149
≥ 2.348	2.98 (1.47–6.03)		2.12 (0.76–5.91)	
< 2.348	1.00		1.00	
**Diastolic blood pressure**	0.98 (0.96–1.00)	0.029	0.97 (0.94–1.00)	0.033
**Heart rate**	1.02 (1.01–1.04)	0.007	1.03 (1.01–1.06)	0.010

## Discussion

In the present study, patients with STEMI and CA125 ≥ 11.48 had a higher risk of mortality at 6 months. CA125 showed a similar ROC curve compared with NTproBNP and hs-CRP to predict mortality. To the best of our knowledge, this is the first study that determined a cutoff value of CA125 for mortality in STEMI.

Over the last few years, biomarkers played an important role in predicting cardiovascular risks. Since atherosclerosis is a multifactorial process, using several markers simultaneously could increase the stratification performance^11^.

Left ventricule impaired function is the most frequent consequence of STEMI and is a strong predictor of mortality. It is caused by myocardial loss or ischemic dysfunction (stunning) and in some cases is worsened by the presence of arrhythmias, valvular dysfunction or mechanical complications. Left ventricle dysfunction may be clinically silent or cause heart failure^12^.

The Killip classification stratifies the severity of acute heart failure complicating myocardial infarction on the basis of the physical signs, and it has been shown to predict mortality. Another way to identify congestion is through biomarkers such as NP. Levels of NP correlate with left ventricular dilatation, remodeling and dysfunction, as well as with death, among patients presenting with acute myocardial infarction. In the first few hours following STEMI, NTproBNP is released as a result of both ischemia and necrosis of myocardial cells. Hence, NTproBNP is considered a good indicator of infarct size and left ventricle function after acute coronary syndrome (ACS)^13–16^.

Hs-CRP plays a key function in the immune response and is involved in the progress and complications of atherosclerosis. Acute phase reactants such as hs-CRP have been proposed as potential indicators of underlying unstable atherosclerotic disease. High circulating hs-CRP levels can significantly and independently predict the occurrence of cardiovascular related death in up to 4 years of follow-up in patients with STEMI^17^.

CA125 is already used in patients with chronic heart failure as a congestion marker. Mechanical stress and inflammation may induce CA125 synthesis from the mesothelial cells of the peritoneum, pleura, and pericardium. It is a prognostic marker of mortality and rehospitalization in this population. In addition, a recent meta-analysis demonstrated that elevated CA125 levels are associated with hospital readmissions and all-cause mortality also in patients admitted with acute heart failure. However, none of the articles included in this meta-analysis evaluated STEMI patients^6,18^.

In patients presenting with ACS, De Gennaro et al. showed that CA125 levels were able to identify patients with pulmonary congestion with higher specificity (97.1 vs. 31.4%), positive predictive value (83.3 vs. 33.3%) and accuracy (83.0 vs. 48.9%) when compared with BNP. However, only 47 patients were included in this study^19^.

Rong et al. have demonstrated that CA125 levels are higher in ACS patients compared to patients with stable coronary disease. In a follow-up of 3 months, 11 of 130 patients suffered from cardiovascular events and 2 died. The patients who had adverse events were more likely to had a significantly higher serum CA125 levels on admission than those without adverse events (20.58 + 16.06 vs. 12.37 + 9.05, *p* = 0.017)^20^.

Separham et al., in 120 male patients with STEMI, evaluated the role of CA125 levels in predicting in-hospital major adverse cardiac events (MACE) including cardiac and non-cardiac death, myocardial infarction, heart failure and the need for revascularization. The mean CA125 levels at admission were 7.99 ± 6.83. Most patients did not present with congestion (91.7%). The MACE was observed in 19 cases (15.8%). Revascularization was the most frequent adverse event, occurring in 11 cases. Overall mortality was 3.3%. They found a significantly higher level of CA125 in patients with MACE (18.92 ± 9.17 vs. 6.01 ± 3.67 < 0.001)^21^.

Our study population was characterized by a group of individuals who presented with a median total ischemic time of 7 h. Almost 18% had pulmonary congestion, 60% had multivessel disease, and angiographic success was obtained in 90%. These factors could explain the higher rate of mortality at 6 months (18%). In the reperfusion era, reported mortality rates range from 4.2% at 1-year to 23.3% at 5 years. Acute heart failure and cardiogenic shock complicate STEMI in 5–19% and in-hospital mortality in these patients is 17–21%^22^.

Patients whose condition exhibits this degree of severity are, in general, poorly represented in trials. Our study represents a real-world scenario. Previous studies with CA125 enrolled a small number of STEMI patients and the majority of patients in Killip I. Our study presented a longer follow-up and evaluated a single “hard” endpoint. Separham et al. used a composite endpoint in which revascularization (“soft endpoint”) accounted for the majority of events. This is the case in which a “soft” clinical endpoint is combined with a “hard” endpoint such as mortality.

The time of CA125 measurement, probably, influences its relation to outcomes. The kinetics of CA125 release from mesothelial cells in STEMI patients is not known. Unfortunately, we measured CA125 levels only at admission. The serial measurement may demonstrate whether the highest or discharge CA125 is a better predictor of mortality than basal levels.

Rong et al. measured CA125 levels on admission and on the fifth day.

CA125 was inversely associated with LVEF only on the fifth day (B =  − 0.842, *p* < 0.001). CA125 was significantly higher in patients who had recurrent heart failure or mortality either on admission or on the fifth day (15.51 ± 10.39 vs. 7.86 ± 5.87, *p* = 0.002). However, an isolated analysis of mortality was not included in their study^20^.

CA125 could replace or be used simultaneously with NTproBNP or hs-CRP for risk stratification in STEMI. It emerges as an alternative biomarker for stratifying heart patients especially because of its wide availability and low cost.

Risk stratification is not a simple process. However, it is important given the increasing number of therapeutic options and a wide spectrum of risk in STEMI patients. Despite the number of possible biomarkers available for risk assessment, it is necessary to prove that it can predict significantly clinical endpoint as mortality at least with the same accuracy comparing to traditional risk tools.

In our analysis, some limitations deserve to be highlighted. This was a single center study with a small number of patients. The biomarkers were measured only at hospital admission, and the level might be dependent on the time elapsed from onset of chest pain. We cannot ignore the fact that factors that influence mortality have not been considered in our analysis (eg, type of stent or treatment at discharge and during the first 6 months).

## Conclusions

This study suggests that elevated CA125 levels might be used to identify patients with STEMI with a higher risk of death at 6 months. CA125 seems to be a similar predictor of mortality compared to NTproBNP and hs-CRP.

## Data Availability

The datasets generated during the current study are available in the https://figshare.com/ repository, named as CA125data.
